# Unmapped short reads from whole-genome sequencing indicate potential infectious pathogens in German Black Pied cattle

**DOI:** 10.1186/s13567-023-01227-0

**Published:** 2023-10-18

**Authors:** Guilherme B. Neumann, Paula Korkuć, Monika Reißmann, Manuel J. Wolf, Katharina May, Sven König, Gudrun A. Brockmann

**Affiliations:** 1 Animal Breeding Biology and Molecular Genetics, Albrecht Daniel Thaer-Institute for Agricultural and Horticultural Sciences, Humboldt-Universität zu Berlin, Berlin, Germany; 2 Institute of Animal Breeding and Genetics, Justus-Liebig-Universität, Giessen, Germany

**Keywords:** Short-read sequencing, illumina sequencing, *Mycoplasma*, DSN, Deutsches Schwarzbuntes Niederungsrind, *Bovine parvovirus 3*

## Abstract

When resequencing animal genomes, some short reads cannot be mapped to the reference genome and are usually discarded. In this study, unmapped reads from 302 German Black Pied cattle were analyzed to identify potential pathogenic DNA. These unmapped reads were assembled and blasted against NCBI’s database to identify bacterial and viral sequences. The results provided evidence for the presence of pathogens. We found sequences of *Bovine parvovirus 3* and *Mycoplasma* species. These findings emphasize the information content of unmapped reads for gaining insight into bacterial and viral infections, which is important for veterinarians and epidemiologists.

**Supplementary Information:**

The online version contains supplementary material available at 10.1186/s13567-023-01227-0.

## Introduction, methods, and results

In livestock genomics, the process of resequencing additional individuals of the same species for which a reference genome exists allows for the investigation of the relationship between sequence variation and diverse phenotypes [1]. Currently, the most widely used technology for resequencing is based on short-read sequencing, where DNA is fragmented into short sequences of 150–300 bp [2]. Raw sequence reads of the resequenced individual are aligned to the reference genome using alignment software such as BWA [3] or Bowtie2 [4]. Sequence reads that cannot be aligned are called unmapped reads and are usually discarded.

Based on the constructed pangenome of 898 cattle from 57 taurine breeds [5], up to 83 Mb corresponding to 3.1% of the cattle genome could not be aligned to the current *Bos taurus* reference genome ARS-UCD1.2, which is derived from a highly inbred Hereford cow [6]. In German Black Pied cattle (DSN, “Deutsches Schwarzbuntes Niederungsrind”), most of the short reads (99.4%) could be mapped to the reference genome, but a small fraction of 0.6% of the reads was left unmapped, on average 2 058 204 reads per animal [7]. The high number of unmapped reads that are discarded during sequencing, especially within large-scale projects such as the 1000 Bull Genomes Project [8], warrants attention towards the analysis of these reads.

Since unmapped reads passed quality control, sequencing errors can be excluded as a reason why the reads do not align to the reference genome. A more likely reason is that the difference between the reference genome and the resequenced individuals is too big. Thus, longer unmapped regions could point to genomic rearrangements such as structural variants [9], which are known to occur in cattle genomes but are difficult to detect with short-read sequencing data [10]. Another reason might be that these unmapped reads could belong to DNA of bacterial or viral organisms that were isolated together with the DNA of the investigated animal from the taken sample. These bacterial or viral DNA might arise from potential pathogenic infection of the sequenced individual or from environmental contamination of the sample.

The sample material from which the DNA was isolated might influence the presence of different bacterial or viral organisms. Samples from ear tissue might be contaminated with soil, which is a habitat for many bacteria. Blood samples are most promising for the detection of pathogenic infections, as they are also used in diagnostics. Recently, a study in birds that investigated unmapped reads obtained from RNA sequencing has detected sequences of different pathogens such as *Plasmodium* and *Trypanosoma* [11]. In Brown Swiss cattle, preliminary analysis of unmapped DNA short reads provided evidence for the presence of *Lactococcus garvieae* [12]. This highlights the potential of unmapped reads obtained from whole-genome sequence data to identify pathogens at the population level, which could be of interest to veterinarians and epidemiologists.

We investigated the unmapped reads resulting from whole-genome sequencing using paired-end 150 bp reads of 302 DSN cattle that could not be aligned to the *Bos taurus* reference genome ARS-UCD1.2. The DSN breed is a small dual-purpose cattle population of 2 500 herdbook cows [13] that is considered a founder population of the high milk-yielding Holstein cattle [14]. DSN has been reported as a robust breed well adapted to pasture conditions. We hypothesized that pathogenic DNA sequences, besides environmentally ubiquitous microorganisms, could be detected in unmapped reads of those sequenced DSN animals.

Whole-genome sequencing data of 302 DSN animals from eight farms in Germany were available from a previous study [7]. The DNA of those samples was obtained from ear tissue (223 samples), blood (42 samples), or sperm (37 samples), and extracted based on a salt-extraction method [15]. Blood was washed in TE buffer for obtaining a white blood cell pellet. The pellet and the ear tissue were handled in the same way by incubating in 180 µL T1 buffer and 20 µL proteinase K [15]. In the case of sperm, 20 µg DTT was added to the lysis reaction. DNA sequencing was performed on the Illumina NovaSeq 150 PE sequencer resulting in an average 18.72-fold coverage. Processing and filtering of sequencing data followed the 1000 Bull Genomes Project guidelines [8] using the *Bos taurus* genome version ARS-UCD1.2_Btau5.0.1Y as reference [6]. This involved trimming low quality bases (qscore < 20) at the beginning and at end of the reads, and filtering out reads with mean qscore < 20 or length < 35 bp using Trimmomatic v.0.9 [16].

Unmapped reads were retrieved and sorted using samtools v.1.12 [17] (*samtools view -u -f 4; samtools sort*), including both single and paired-end unmapped reads. Per animal, an average 2 058 204 (SE 180 007) unmapped reads were retrieved out of 348 148 174 (SE 2 669 357) sequence reads that passed quality control. The numbers of unmapped reads per animal ranged from 144 848 to 40 734 422 across the 302 sequenced DSN animals.

Assembly of unmapped reads was performed for each animal using Abyss v.1.5.2 [18]. Kmer sizes from 50 to 90 (maximum kmer size in Abyss) were tested for 30 randomly selected animals (Additional file 1). K-mer refers to a sequence of contiguous nucleotides. That means each unmapped sequence read is used to generate sequences of contiguous nucleotides in a sliding window, where k is the window size in bases. Kmer size = 90 was used for generating contigs since they produced on average the longest contigs and thereby the lowest number of contigs. Based on the information of paired-end reads, gaps between adjacent contigs were linked to create scaffolds (Additional file 2). On average, 11 354 (SE 607) scaffolds with an average length of 779 bp (SE 72 bp) were generated per animal.

Scaffolds with a minimum length of ≥ 500 bp were compared individually to the reference and representative genomes of viruses, prokaryotes, and eukaryotes as obtained from NCBI [19] (2022/07/18) using Blast + v.2.12.0 software for nucleotides [20]. The NCBI eukaryotes database included the *Bos taurus* reference genome which is based on a Hereford cow, the *Bos indicus* assembly which is based on a Nellore bull, and the hybrid *Bos indicus x Bos taurus* assembly (GCA_003369695.2 UOA_Brahman_1) which is an F1 cross between an Angus sire (*Bos taurus*) and a Brahman dam (*Bos indicus*) [21]. From the F1 cross, only the maternal haplotypes of the *Bos indicus* Brahman breed were available in the database of representative genomes. Each Blast result was sorted according to a matching score, so-called E-value. The smaller the E-value, the stronger the statistical significance. The five top-scoring results from each database of representative genomes were kept (lowest E-value after filtering for query coverage ≥ 95% and identity percentage ≥ 95%). From those top-scoring results of the three databases, only the result with the longest coverage and highest identity percentage was considered.

Scaffolds mapping to the same species were reported as assemblies. When multiple scaffolds mapped to the same species, the corresponding scaffold lengths were summed up to represent the assembly length. Only assemblies covering at least 10% of the investigated genomes of viruses, prokaryotes, and eukaryotes were considered for the further analysis. K-mer depth was calculated as the number of overlapping k-mers per base in the final assemblies. Genes occurring in the assemblies blasted to the *Bos* species were retrieved using the annotation from NCBI. The presence of pathogenic assemblies was validated using PCR. Primers were designed using NCBI’s Primer-BLAST software [22].

In the assemblies from unmapped sequence reads of 116 DSN animals, DNA sequences of viruses and bacteria were detected covering at least 10% of the respective viral or bacterial genome. This included the detection of DNA sequences of six viruses and eleven bacterial species. In the unmapped sequence information, the following DNA sequences of viruses were found: 16 DSN animals contained DNA sequences of the *Deformed wing virus*, four DSN animals contained the *Bovine parvovirus 3*, four DSN animals contained the *Brassica yellows virus*, three DSN animals contained the *Stenotrophomonas phage SMA7*, one DSN animal contained the *Helicoverpa armigera densovirus* and one DSN animal contained the *PreXMRV-1*. These are all small viruses with a genome size ranging from 5.33 kb to 10.14 kb (Additional file 3). From this list, the only virus known to frequently infect cattle is the *Bovine parvovirus 3*. The DNA sequence of *Bovine parvovirus 3* was found in animals that were sequenced from either ear tissue or blood samples. The animals were kept in three different farms and ear tissue or blood were collected in different years (Table 1).

**Table 1:** **Animal sample information of four DSN animals whose unmapped sequencing data showed the presence of** ***Bovine parvovirus 3***. The assembly information includes the length of the assemblies generated from unmapped reads that could be mapped to the *Bovine parvovirus 3* genome (full genome size = 5.33 kb), the genome coverage, and the average k-mer depth of the respective assembly.

Animal sample information					Assembly information			
Sex	Birth year	Farm	Tissue	Collection (year/month)	Number of scaffolds	Length (kb)	Genome coverage (%)	K-mer depth
Bull	2015	1	Blood	2015/10	1	5.32	99.8	41.47
Cow	2015	2	Ear	2017/08	2	5.20	97.6	6.64
Cow	2014	3	Ear	2017/08	1	5.50	100.0	11.25
Cow	2015	2	Ear	2017/08	1	0.61	11.4	2.41

With regard to bacterial species, the unmapped sequence information of 37 DSN animals contained DNA sequences of *Achromobacter insuavis*, while *Variovorax gossypii* was found in 18 DSN animals, *Roseateles aquatilis* in 12 DSN animals, *Bosea lupini* in nine DSN animals, *Mycoplasma wenyonii* str. Massachusetts in four DSN animals, *Pseudomonas paracarnis* in three DSN animals, *Candidatus* Mycoplasma haemobos in two DSN animals, and *Delftia acidovorans*, *Pseudomonas carnis*, *Pseudomonas lactis*, and *Pseudomonas salmasensis* each in one DSN animal (Additional file 4). Of those, only the *Mycoplasma* species naturally infect cattle, while the *Pseudomonas* species are known to proliferate in stored beef and milk. The other bacteria are found primarily in soil. Interestingly, *Mycoplasma* species were only detected in blood samples, and *Pseudomonas* only in sperm samples (Table 2). For one cow, DNA sequences of both *Candidatus* Mycoplasma haemobos and *Mycoplasma wenyonii* were detected, covering their genome with 11.29% and 33.71%, respectively.

**Table 2:** **Animal sample information of six DSN animals whose unmapped sequencing data showed the presence of** ***Mycoplasma*** **species**. The assembly information includes the length of the assemblies generated from unmapped reads that could be mapped to the *Mycoplasma* species *Candidatus* Mycoplasma haemobos (full genome size = 935 638 bp) and *Mycoplasma wenyonii* (full genome size = 650 228 bp), the respective genome coverage, and the average k-mer depth of the respective assembly.

Mycoplasma species	Animal sample information					Assembly information			
	Sex	Birth year	Tissue	Farm	Collection (year/month)	Number of scaffolds	Length (kb)	Genome coverage (%)	K-mer depth
*Candidatus* Mycoplasma haemobos	Cow	2013	Blood	1	2015/10	168	670.04	71.6	4.90
	Cow	2014	Blood	1	2015/10	126	105.59	11.3	2.34
*Mycoplasma wenyonii*	Cow	2014	Blood	1	2015/10	27	218.93	33.7	4.06
	Bull	2014	Blood	1	2015/10	2	179.86	27.7	27.21
	Bull	2015	Blood	1	2015/10	2	179.82	27.7	161.85
	Bull	2015	Blood	1	2016/11	7	173.58	26.7	1768.83

The presence of *Candidatus* Mycoplasma haemobos and *Mycoplasma wenyonii* was validated using PCR (Additional file 5). Among four animals detected with *Mycoplasma wenyonii* in the unmapped reads, three had enough DNA for a validation PCR. In the DNA of all three animals PCR fragments of *Mycoplasma wenyonii* could be amplified using primers targeting the 23S rRNA region. The two animals detected with *Candidatus* Mycoplasma haemobos also showed strong amplification for *Candidatus* Mycoplasma haemobos in the PCR, but a weak amplification was also observed in two out of three negative controls. The PCR primers were also flanking the 23S rRNA. No amplification was obtained for *Bovine parvovirus 3* although two different sets of primers were tested (Additional file 5).

The unmapped reads of the investigated DSN animals showed no evidence for the presence of foreign eukaryotic DNA, with assemblies covering > 10% of the foreign eukaryotic genomes. However, smaller assemblies obtained from all 302 DSN animals could be mapped to different *Bos* species including *Bos indicus*, *Bos indicus x Bos taurus*, and *Bos mutus*. The average assembly length of these smaller assemblies was 341.3 kb (SE 6.9 kb) corresponding to 0.01% of the cattle reference genome. These assemblies spanned genomic regions of 388 genes, which were covered by assemblies from unmapped sequence information of on average 19.9% of the 302 DSN animals. Around 80% of the DSN animals had sequences covering ten genes. These ten genes are located on eight chromosomes: *THSD7B* (Thrombospondin type 1 domain containing 7B) on chromosome 2, *VTCN1* (V-set domain containing T cell activation inhibitor 1) on chromosome 3, *SAMD9* (Sterile alpha motif domain containing 9) on chromosome 4, *LOC109559963* (rho GTPase-activating protein 20-like) on chromosome 6, *RASGRF2* (Ras protein specific guanine nucleotide releasing factor 2) on chromosome 7, *NDUFAF2* (NADH:ubiquinone oxidoreductase complex assembly factor 2) on chromosome 20, *LOC113881290* (heat shock 70 kDa protein 1A) and *PKHD1* (PKHD1 ciliary IPT domain containing fibrocystin/polyductin) on chromosome 23, and *TCERG1L* (transcription elongation regulator 1 like) and *SUFU* (SUFU negative regulator of hedgehog signaling) on chromosome 26.

## Discussion

In this study, we identified pathogenic DNA sequences of *Mycoplasma* species and *Bovine parvovirus 3* based solely on usually discarded unmapped reads from whole-genome sequencing data. These could point to bovine pathogens, but also to potential biological contaminations. The in silico detected *Mycoplasma* on the unmapped reads could be validated using PCR, while PCR validation could not be provided for *Bovine parvovirus 3*. Furthermore, we provide evidence that the detection of pathogens depends on the tissue from which the DNA was extracted. For example, *Mycoplasma* was detected only when blood and not sperm or ear tags were used for DNA extraction.

The reliability for detecting the correct bacterial and virus species in the sequence information of DSN was built upon assemblies that mapped to more than 10% of the representative pathogen genome, and on parameters such as contiguity (number of scaffolds) and k-mer depth (coverage of k-mer in assemblies). Although less reliable, shorter assemblies covering less than 10% of the pathogen genome indicated the potential presence of *Mycoplasma* in more DSN animals and the presence of *Bovine gammaherpesvirus 6* (data not shown).

Infection with the *Bovine parvovirus 3*, which was found in the unmapped sequence information of four DSN animals, has been linked so far only to bovine respiratory disease [23, 24]. However, cases of diarrhea in neonatal calves and respiratory and reproductive diseases in adult cattle have been associated with other bovine parvoviruses [25]. More recently, viral metagenomics analyses revealed that *Bovine parvovirus 3* is the most abundant virus in fetal bovine serum on almost all continents [26]. The other viruses detected in the unmapped sequence information of DSN are environmental viruses which do not infect cattle.

The detection of *Mycoplasma* in DSN was expected, since the presence of this pathogen is known and linked to mastitis infections [27, 28]. DSN cows with mastitis are routinely tested for *Mycoplasma* together with other bacterial infections. However, the classification of *Mycoplasma* at species level is novel. Recently, *Candidatus* Mycoplasma haemobos has been identified in cattle in Northern Germany for the first time [29]. This is the region where most of the DSN animals come from. In the same study, infections with *Mycoplasma wenyonii* were reported [29]. Coinfection with both *Mycoplasma* species have been reported in different places [30, 31]. Interestingly, using DNA sequence analysis of unmapped reads, we could also identify one animal that was detected with DNA of both *Mycoplasma* species. Furthermore, infections with *Candidatus* Mycoplasma haemobos and *Mycoplasma wenyonii* had been detected in blood only [29–33].

Some pathogens were detected only in DNA extracted from specific tissues. DNA sequences of *Mycoplasma*, for example, were detected only in DNA extracted from blood, while soil bacteria such as *Variovorax gossypii* [34] and *Bosea lupini* [35] were detected mainly in DNA extracted from ear tissue clips, pointing to potential sample contamination. *Pseudomonas* has been reported before in bovine raw milk and refrigerated beef [36, 37]. In our study, we detected this species only in unmapped sequence information obtained from sperm samples.

This study presents an analysis of unmapped reads obtained from cattle previously sequenced. For further validation of microorganisms that are not typically infectious in cattle, it would also be necessary to validate their occurrence via PCR or direct detection which would serve as evidence of biological contamination. Since contamination via ubiquitous microorganisms varies among different tissue types, we emphasize the importance of collecting tissue-specific metadata, ensuring this valuable information is available for drawing conclusions.

Furthermore, the lack of *Bos taurus* results from the Blast analysis was anticipated, as the assemblies were generated from sequence reads that did not initially map to the *Bos taurus* reference genome. The fact that some assemblies from the unmapped reads of DSN could be mapped to other *Bos* species suggests that the current *Bos taurus* reference genome cannot capture all of the sequence variation found in DSN. Repetitive regions such as simple repeats, tandem repetitions, microsatellites, and even short and long interspersed nuclear elements, and structural variations such as insertions, and tandem duplications are huge challenges in sequence alignment [38]. For the identified genes that were covered by 80% of the DSN assemblies, observations about structural variants in the Ensembl database release 109 were found. For instance, two deletions (esv4015120 and esv4018365) and one tandem duplication (esv3899898) are known for *VTCN1*, seven deletions (esv3896092, esv4013846, esv4018847, esv4013542, esv4019566, esv4014539, and esv3896170) and one insertion (nsv810851) for *RASGRF2*, and two deletions (esv3894860 and esv4012358), two inversions (esv3897812 and esv3897810), and one tandem duplication (esv3899664) for *PKHD1*. Therefore, DNA sequences that map to other *Bos* species than the Herford reference genome for *Bos taurus* point to potential structural variations occurring in DSN which should be better characterized in the future with other mapping strategies, such as applied in the pangenome consortium [39] and the utilization of long-read sequencing data by the long-read consortium for cattle [40].

In conclusion, we revealed for the first time the presence of *Candidatus* Mycoplasma haemobos and *Mycoplasma wenyonii* in DSN by using information from unmapped reads of short-read sequencing data. The unmapped short reads could also provide evidence for infections if other data is lacking. Further investigations of unmapped short reads are needed in order to elucidate the reliable use of such information which can be very useful for veterinarians and epidemiologists, in particular to understudied animal health in populations.

### Supplementary Information

**Additional file 1: Average number of contigs and N50 of assembled unmapped reads from 30 randomly selected DSN animals.** K-mer sizes from 50 to 90 were tested.

**Additional file 2: Genome assembly scheme.** (1) Unmapped sequence reads (~150bp) were first extracted from alignment files. (2) K-mers were generated from unmapped reads with sliding window size of 90 bp and step size of 1 bp, producing around 61 k-mers per unmapped read. (3) Based on the k-mers similarities and on the de-Bruijn graph algorithm implemeted by Abyss, contigs were formed. (4) Contigs were linked together whenever possible based on the paired-end reads used to generate the k-mers. (5) The resulting assemblies for each species consist of scaffolds that were sorted according to their blast results

**Additional file 3: Average assembly and animal sample count information for detected viruses in 27 DSN cattle.** Those were Deformed wing virus (DWV), Bovine parvovirus 3 (BP3), Brassica yellows virus (BYV), Stenotrophomonas phage SMA7 (SMA7), and Helicoverpa armigera densovirus (HAD). Arithmetic means are given for assembly size, k-mer depth, number of scaffolds, and genome cover, with standard errors given in parenthesis.

**Additional file 4: Average assembly and animal sample count information for detected bacteria in DSN cattle.**  Arithmetic means are given for assembly size, k-mer depth, number of scaffolds, and genome cover, with standard errors given in parenthesis.

**Additional file 5: PCR results.**

## Data Availability

Publicly available datasets were analyzed in this study. This data can be found here: https://www.ebi.ac.uk/ena/browser/view/PRJEB45822.
