# Supplementary figures and images for: Unmapped short reads from whole-genome sequencing indicate potential infectious pathogens in German Black Pied cattle
Source: Vet Res. 2023 Oct 18;54:95. doi: 10.1186/s13567-023-01227-0 (PMC10585868; doi:10.1186/s13567-023-01227-0)

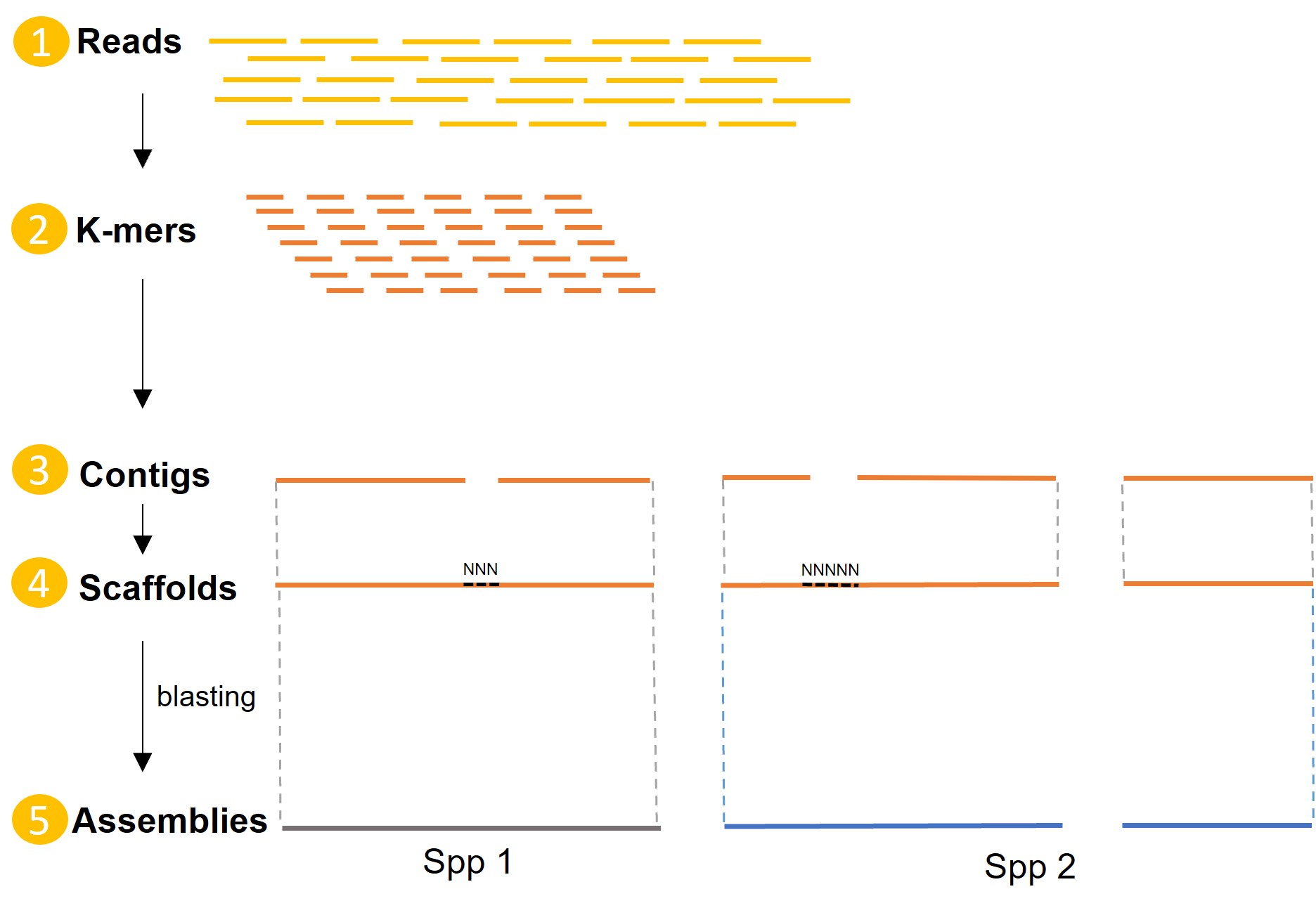

Supplement: Supplementary file 2 — Additional file 2: Genome assembly scheme. (1) Unmapped sequence reads (~150bp) were first extracted from alignment files. (2) K-mers were generated from unmapped reads with sliding window size of 90 bp and step size of 1 bp, producing around 61 k-mers per unmapped read. (3) Based on the k-mers similarities and on the de-Bruijn graph algorithm implemeted by Abyss, contigs were formed. (4) Contigs were linked together whenever possible based on the paired-end reads used to generate the k-mers. (5) The resulting assemblies for each species consist of scaffolds that were sorted according to their blast results [file 13567_2023_1227_MOESM2_ESM.jpg]
