# Supplementary material for: Unmapped short reads from whole-genome sequencing indicate potential infectious pathogens in German Black Pied cattle
Source: Vet Res. 2023 Oct 18;54:95. doi: 10.1186/s13567-023-01227-0 (PMC10585868; doi:10.1186/s13567-023-01227-0)
Supplement: Supplementary file 5 — Additional file 5: PCR results. [file 13567_2023_1227_MOESM5_ESM.pdf]

PCR for the detection of:

Mycoplasma wenyonii: forward 5’-GAGGGCACTCTTTCTCCCGA-3’, reverse 5’-GCGATGGCTGAGAGAGCCAG-3’. Expected fragment length: 182 bp.

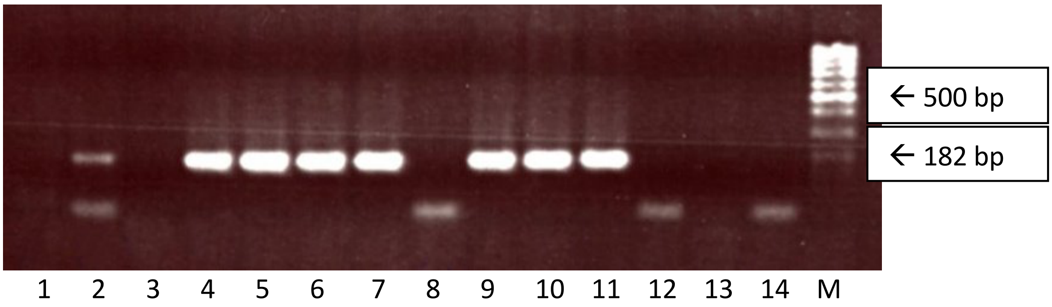

Position	Lab Code	Whole-genome sequence information on pathogen infection	DNA preparation replicate	Tissue
1	BU131	Genome coverage: 34%, K-mer depth: 4.1	A	ear
2	BU131	Genome coverage: 34%, K-mer depth: 4.1	A	blood
3	empty	-	-	-
4	Bu131	Genome coverage: 34%, K-mer depth: 4.1	B	blood
5	Bu118	Genome coverage: 28%, K-mer depth: 27.2	A	blood
6	Bu118	Genome coverage: 28%, K-mer depth: 27.2	B	blood
7	Bu118	Genome coverage: 28%, K-mer depth: 27.2	C	blood
8	Bu139	Genome coverage: 27%, K-mer depth: 1768.8	A	Sperm, original DNA from blood not available
9	Bu120	Genome coverage: 28%, K-mer depth: 161.8	A	blood
10	Bu120	Genome coverage: 28%, K-mer depth: 161.8	B	blood
11	Bu120	Genome coverage: 28%, K-mer depth: 161.8	C	blood
12	Bu121	-	A	blood, control
13	Bu122	-	A	blood, control
14	Bu123	-	A	blood, control

***Candidatus* Mycoplasma haemobos**: forward 5'- GGCAGGCTATGAAGGACGTG-3', reverse 5'-TCACTTCACGAGGTTCGCTT-3'. Expected fragment length: 156 bp.

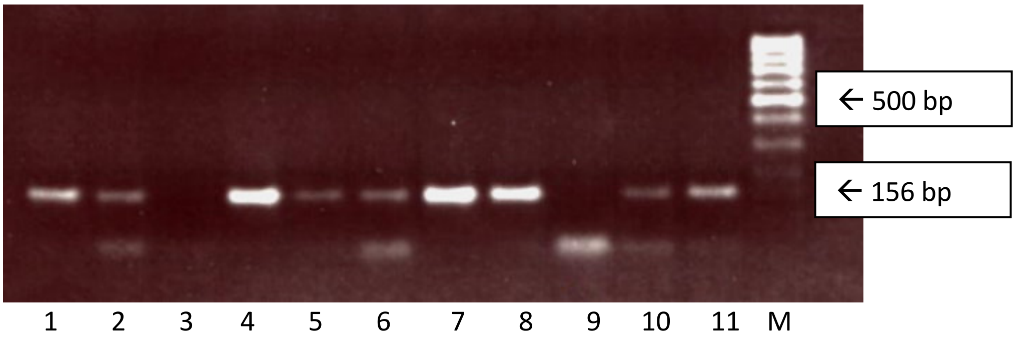

Position	Lab Code	Whole-genome sequence information on pathogen infection	DNA preparation replicate	Tissue
1	BU131	Genome coverage: 11%, K-mer depth: 2.3	A	ear
2	BU131	Genome coverage: 11%, K-mer depth: 2.3	A	blood
3	empty	-	-	-
4	Bu131	Genome coverage: 11%, K-mer depth: 2.3	B	blood
5	Bu126	Genome coverage: 72%, K-mer depth: 4.9	A	ear
6	Bu126	Genome coverage: 72%, K-mer depth: 4.9	A	blood
7	Bu126	Genome coverage: 72%, K-mer depth: 4.9	B	blood
8	Bu126	Genome coverage: 72%, K-mer depth: 4.9	C	blood
9	Bu121	-	A	blood, control
10	Bu122	-	A	blood, control
11	Bu123	-	A	blood, control

***Bovine parvovirus* 3:** forward 5'-GCGATACGTGGTTGCCTGTG-3', reverse 5'-ACTTCGCCCTCGAATACCCG-3'. Expected fragment length: 163 bp.
Forward 5'-TCTCCCACTCCCGTATTAAT-3', reverse 5'-AATCTCCTCCGCTTTCAAAA-3'. Expected fragment length: 165 bp.

No amplification observed for both primer sets.
